# The populism-euroscepticism nexus in a contested Europe: The EUPopLink COST Action

**DOI:** 10.12688/openreseurope.22680.1

**Published:** 2026-01-26

**Authors:** Ioannis Andreadis, Sofia Vasilopoulou

**Affiliations:** 1 School of Political Sciences, Aristotle University of Thessaloniki Faculty of Economic and Social Sciences, Thessaloniki, Makedonia Thraki, 54124, Greece; 2 King's College London Department of European & International Studies, London, England, WC2B 6NR, UK

**Keywords:** Populism, Euroscepticism, European Union (EU), Polycrisis, Ideological Cleavages

## Abstract

The EUPopLink COST Action (CA23102) addresses the complex and changing relationship between populism and Euroscepticism in contemporary Europe. While often viewed as "two sides of the same coin," the nexus between these two phenomena is contingent and strategic rather than deterministic. Not all populists are Eurosceptic, and Euroscepticism is not always populist in nature. This publication synthesizes current scholarly debates, highlighting how the European Union (EU) is frequently framed as the populist "other", i.e., a remote, technocratic elite standing in opposition to the "pure people". The nature of this opposition varies significantly across the ideological spectrum, e.g. right-wing populism targets the EU primarily through a "traditionalist-authoritarian-nationalist" (TAN) lens, viewing it as a threat to national sovereignty and cultural identity, while left-wing populism critiques the EU based on socio-economic cleavages, challenging neoliberal governance and austerity while often advocating for a more democratic "Social Europe". The analysis further explores how the polycrisis era—marked by financial instability, the COVID-19 pandemic, and geopolitical conflict—has led to a sophisticated two-level strategy among populist actors. This involves increased institutional pragmatism coupled with radicalized, opportunistic communication, particularly on social media. Finally, the publication outlines the EUPopLink mandate, which seeks to standardize conceptual frameworks and generate an unprecedented body of comparative data through forthcoming country reports. By linking the supply and demand sides of electoral competition, the project aims to provide policymakers and scholars with the tools necessary to mitigate the negative consequences of these disruptive political forces.

## Lay Summary

**Understanding the Link Between Populism and Euroscepticism**

Across Europe, many people feel disconnected from the way the European Union (EU) works. This has led to the rise of populism (a "people versus the elite" style of politics) and Euroscepticism (criticism of the EU). While these two often go together, they are not the same. If we want to protect democratic stability, we need to understand how they interact and how leaders use these feelings to win support.

**The "Elite" vs. "The People":** Populist leaders often use the EU as a "punching bag". Because EU decisions can feel distant or complicated, it is easy for leaders to frame "Brussels" as a corrupt elite that ignores the "ordinary person". However, different political groups do this for different reasons:

- **The Radical Right:** They often focus on national identity and culture. They worry the EU is taking away a country’s control over its own borders and values.
- **The Radical Left:** They focus on redistribution and fairness. They often criticize the EU for promoting economic policies that they believe hurt workers and instead favor big banks or corporations.

**A Changing Strategy** The relationship between these two phenomena is constantly changing, especially during crises like the COVID-19 pandemic or the war in Ukraine. Interestingly, many populist parties have become more "pragmatic" or professional when they are in power. However, they still use radical and emotional language on social media to mobilise their voters.

**What is EUPopLink doing?** The EUPopLink project is a major international effort to study these trends across many different countries. By creating a shared set of definitions and collecting data across Europe, the project aims to help researchers and policymakers understand why people are unhappy and how to respond in a way that strengthens democracy.

## Introduction: An intertwined but unsettled relationship

### The central puzzle

In the landscape of contemporary European politics, populism and Euroscepticism have emerged as two of the most significant and disruptive forces, fundamentally challenging the institutional order of the European Union (EU) and its member states. Empirically, the two phenomena are deeply intertwined; populist political actors are frequently the most vocal critics of European integration, and citizens with populist attitudes often express high levels of Euroscepticism. This overlap has underpinned a perception, both in public discourse and in early academic analyses, that populism and Euroscepticism are two sides of the same coin. Yet, this apparent alignment masks a complex and unsettled reality. Despite their frequent co-occurrence, there remains little agreement on how populism and euroscepticism relate to each other both conceptually and empirically.

The relationship is contingent, not deterministic; while powerful theoretical and political logics frequently drive the two together, the connection is neither automatic nor uniform ( Andreadis *et al.*, 2022); not all populists are Eurosceptic, and not all Euroscepticism is populist. This analytical ambiguity represents a significant obstacle to understanding patterns of political discontent in Europe, analyzing their drivers and formulating effective responses.

Analyzing the nexus between the two phenomena has profound implications for the stability and future of the European project. The rise of political actors, primarily the far right, who strategically fuse populist and Eurosceptic appeals has been linked to increased political polarization, the erosion of democratic norms, and a decline in institutional trust ( Macková *et al.*, 2025). Without a rigorous framework for analyzing how these phenomena interact, reinforce, or occasionally diverge from one another, scholars and policymakers alike will remain ill-equipped to grasp the nature of the challenge and to identify potential avenues for mitigating its negative consequences. The intellectual starting point for any academic inquiry must therefore be one of disaggregation. The very frequency with which the terms are used interchangeably has led to a degree of conceptual stretching, obscuring crucial distinctions between a political ideology, logic, strategy or style (populism) and a more specific policy orientation (Euroscepticism). Populism operates at a higher level of conceptual abstraction as a broader political concept concerning the functioning of representative democracy, whereas Euroscepticism is a concrete stance on the specific issue of European integration and its various dimensions ( Rooduijn & van Kessel, 2019). By treating their linkage as a subject for empirical investigation rather than an a priori assumption, it becomes possible to ask more precise and meaningful questions: Under what conditions does populism translate into Eurosceptic policy agendas? How do different ideological traditions shape the nature of this nexus? And how do populist actors strategically deploy Eurosceptic themes in response to evolving political and economic crises?

### The EUPopLink mandate

It is precisely this analytical challenge that the COST Action "Linking euroscepticism and populism: causes and consequences" (EUPopLink) is designed to address. As the most comprehensive and coordinated international effort to date, EUPopLink's main objective is to analyze the interplay between Euroscepticism and populism, to identify its causes and consequences and how the negative consequences can be mitigated, using and combining existing datasets and exploring both the supply and the demand side of electoral competition. This mandate recognizes that progress in the field requires a multi-faceted approach that is comparative, methodologically innovative, and longitudinal. It also acknowledges the contemporary political environment: populist and Eurosceptic leaders are themselves increasingly cooperating across borders, learning from one another's experiences and forming transnational networks. For the scholarly community to keep pace, let alone provide meaningful analysis, a similar level of purposive international academic cooperation is not just beneficial, but essential.

### Deconstructing the core concepts: Populism and Euroscepticism

A precise understanding of the populism-Euroscepticism nexus requires a firm grasp of its constituent parts. Both concepts have been the subject of extensive scholarly debate, evolving from broad descriptors into more refined and multi-dimensional analytical frameworks. While a consensus has emerged viewing populism as a normative distinction centered on a moralized antagonism between the pure people and the corrupt elite, significant debates persist regarding its precise nature—whether it is a "thin-centered ideology", a "discursive" logic, or a political "style" or "strategy". Likewise, the study of Euroscepticism has moved beyond a simple criticism of European integration to a complex field featuring multiple typologies, such as the distinctions between "hard" and "soft" opposition, "policy" and "polity" critiques, or ideologically distinct "left-wing" and "right-wing" variants.

To navigate this complexity and provide a stable foundation for its research, the EUPopLink COST Action (CA23102) has produced a comprehensive Theoretical Framework, which provides an in-depth review of these conceptual debates and establishes the specific, nuanced working definitions used by the project. It outlines the project's subcategories of populism, e.g., populist radical right, left-wing populists, and valence populists, and adopts a multi-faceted approach to Euroscepticism, analyzing its object, intensity, and nature. This introduction to the EUPopLink project draws directly upon that foundational framework to analyze the nexus between the two phenomena.

### Argument and structure

This publication serves as a foundational review of the field, synthesizing the state of the art and charting a forward-looking research agenda that aligns with the EUPopLink’s mission. Its central argument is that the populism-Euroscepticism nexus is not a static or monolithic phenomenon. Rather, it is a dynamic, strategic, context-specific, and ideologically variegated relationship that has significantly developed over time, particularly in the polycrisis era that has defined European politics since the late 2010s. In the next section, we analyze the theoretical and empirical nature of the nexus itself, exploring both the symbiotic logic that often binds the two phenomena and the conditions under which they diverge. Finally, we argue that the EUPopLink project, through its specific conceptual and empirical deliverables, represents the primary and essential mechanism for executing this agenda and advancing the discipline.

## The populism-Euroscepticism nexus: A symbiotic but contingent relationship

Having established the conceptual foundations of populism and Euroscepticism as distinct phenomena, the analysis now turns to the nature of their interaction. It varies significantly across the ideological spectrum, specific country contexts, and is subject to strategic calculations by political actors.

### The EU as the populist 'Other'

The primary theoretical linkage between populism and Euroscepticism stems from the populist worldview itself. The core populist narrative requires a clear antagonist, i.e., an allegedly remote, powerful, and unresponsive 'elite' against which 'the people' can be mobilized. Populism predominantly involves an anti-establishment discourse. If the EU constitutes the established structure within which the lives of European citizens evolve, it is hardly surprising that it is often portrayed as a primary embodiment of the elite that populism seeks to challenge (see Stavrakakis *et al.*, 2017). As a result, in the European context, the EU often serves as a leading symbol of this elite 'other'. Its institutions are distant from many citizens, its decision-making processes are complex and often perceived as technocratic and non-transparent, and it is frequently associated with a democratic deficit. This institutional structure makes the EU a natural and highly salient target for populist actors seeking to frame politics as a struggle between the common sense of the average person and the out-of-touch machinations of a supranational establishment.

This dynamic is not merely theoretical; it is a central feature of populist discourse across the continent. Political leaders like Viktor Orbán in Hungary and Jarosław Kaczyński in Poland have built their political projects around a narrative that explicitly equates the EU with "the corrupt elite". In their rhetoric, "Brussels" is not just a location but a symbol of an external power that stands in conflict with the "pure people", the Hungarians and Poles, and acts against the principle of popular sovereignty ( Csehi & Zgut, 2021; Mach, 2022). This strategy involves a three-step rhetorical model: (1) constructing an external threat (the EU and its liberal values), (2) demonizing domestic political opponents as collaborators with this external threat, and (3) positioning themselves as the sole authentic defenders of the nation ( Bergmann, 2025). The EU's perceived imposition of policies on sensitive issues like migration or cultural values provides a constant stream of material for right-wing populist leaders to frame as an elite assault on the national way of life. On the other hand, persistent economic inequalities provide similar opportunities for left-wing populist leaders. As a result, both right-wing and left-wing populist leaders are able to fuel both anti-elite and anti-EU sentiment simultaneously.

### Ideological divergence in the populist-Euroscepticism nexus

The general tendency for populism to target the EU as an elite entity does not mean the nexus is ideologically uniform. A critical advance in the field has been the recognition that the *reasons* for this opposition, and thus the character of the nexus itself, differ profoundly between the left and right ends of the political spectrum. These differences are rooted in distinct definitions of 'the people' and 'the elite' and are animated by fundamentally different social cleavages.

The **right-wing populism-Euroscepticism nexus** is primarily animated by what is often termed the "traditionalist-authoritarian-nationalist" (TAN) cleavage. Here, 'the people' are defined in cultural, ethnic, or national terms—as the authentic members of a historic national community. 'The elite' is a cosmopolitan, liberal establishment, both at the national and EU level, accused of undermining this community through the promotion of multiculturalism, open borders, and progressive social values ( Mach, 2022). The core grievance is the perceived threat to national sovereignty and cultural identity. Their vision for Europe's future is typically a "Europe of Nations," a looser confederation of sovereign states stripped of supranational political power ( Vasilopoulou, 2011; Vasilopoulou, 2018). Consequently, their Eurosceptic critique targets EU policies related to immigration (Schengen Area), asylum, and the jurisdiction of European courts, which are seen as eroding the nation's ability to control its borders and maintain its cultural homogeneity ( Pirro *et al.*, 2018). Among right-wing voters, Euroscepticism tends to be related to cultural and sovereignty concerns ( van Elsas & van der Brug, 2015).

The **left-wing populism-Euroscepticism nexus**, in contrast, is rooted in a socio-economic cleavage. 'The people' are defined in class terms, as ordinary citizens, workers, and the economically precarious. 'The elite' is a neoliberal establishment, comprising financial institutions, multinational corporations, and the EU bodies (like the European Commission and the European Central Bank) that enforce their agenda. The core grievance is economic injustice, inequality, and the dismantling of the welfare state ( Wagner, 2021). Their Eurosceptic critique therefore focuses on EU economic governance, such as the Stability and Growth Pact, fiscal austerity measures imposed during the Eurozone crisis, and trade agreements perceived to favor capital over labor. Unlike their right-wing counterparts, their goal is often not to dismantle Europe but to create "another Europe"—a "Social Europe" ( Baute *et al.*, 2019) with stronger democratic accountability, social protections, and a commitment to reducing inequality. Accordingly, among left-wing citizens Euroscepticism is primarily driven by socio-economic considerations ( van Elsas & van der Brug, 2015).

### De-linking the concepts: When populism and euroscepticism diverge

Despite the powerful logics binding them, it is crucial to recognize that the Populism-Euroscepticism nexus remains contingent. The link is not inevitable, and several factors can lead to its weakening or absence. Research has shown that populism and Euroscepticism "do not always coexist" ( Pirro & Taggart, 2018; Rooduijn & van Kessel, 2019). Criticism of European integration is not always framed in a populist manner; it can be advanced on technocratic, legal, or purely pragmatic grounds.

Conversely, and perhaps more surprisingly, populist actors are not always Eurosceptic. In some instances, populists may view the EU as a potential ally or a useful lever against *domestic* elites. A populist party in opposition might appeal to EU institutions to sanction a corrupt national government, for example. Furthermore, for some populist parties, the issue of European integration may simply not be salient. They may choose to focus their anti-elite rhetoric on purely domestic issues like corruption, crime, or unemployment, viewing the EU as a secondary concern for their voters.

The strategic choice of whether to activate a Eurosceptic discourse depends on the national political context, the salience of European issues, and the party's ideology and/or electoral calculations. Recent scholarship further reinforces this point, arguing that populism is only "one possible discursive articulation to mobilize the contestation of the EU" ( Roch, 2023). Radical parties can and do build their Euroscepticism on other hegemonic discourses, such as nationalism or specific economic ideologies, without necessarily employing a populist people-versus-elite frame ( Roch, 2023). This underscores the analytical necessity of treating the two concepts as distinct and their relationship as a variable to be explained, not a constant to be assumed.

### The nexus in a polycrisis era

The relationship between populism and Euroscepticism is not static; it adapts to the prevailing political environment. For almost two decades, the EU has been through a "polycrisis", i.e., a series of cascading and overlapping crises including the Eurozone financial crisis, the massive influx of asylum seekers, the United Kingdom's departure from the EU, the COVID-19 pandemic, the war in Ukraine, the subsequent energy crisis, and persistent inflation ( Arce *et al.*, 2024). This new context has prompted a significant strategic evolution in how populist actors engage with the EU, a trend clearly visible in the most recent academic literature. A more sophisticated, two-level strategy has emerged. At the institutional level, many populist parties have adopted a more pragmatic stance to maintain political viability. Simultaneously, at the public-facing, communicative level, they have become more opportunistic, leveraging the constant stream of crises to fuel voter discontent through emotionally resonant anti-EU narratives.

While populist actors have become more institutionally pragmatic, they have simultaneously become more communicatively radical and opportunistic. The polycrisis era has provided a fertile ground for populist mobilization by creating a continuous stream of events that can be framed as evidence of EU failure. Recent scholarship emphasizes that these crises are not merely a passive backdrop but are actively instrumentalized as "narrative devices" to fuel Euroscepticism ( Carpenter *et al.*, 2024). Such crises, including the pandemic response, energy policy, or support for Ukraine, may offer further opportunities for populist actors to construct "totalizing narratives" that portray the EU as incompetent, overbearing, or acting against the interests of the “ordinary people” ( Carpenter *et al.*, 2024).

This strategy is particularly effective on social media, where political actors can directly deliver emotionally charged, conflict-oriented, and visually striking content to voters ( Macková *et al.*, 2025). Studies of communication in the Visegrád countries during the 2024 European Parliament elections show that populist actors were the most frequent users of anti-EU messaging, consistently employing an anti-elitist frame accompanied by emotional tools like fear appeals and patriotic symbols ( Macková *et al.*, 2025).

## Conclusion: Synthesizing knowledge and charting the path forward with EUPopLink

This introduction has sought to demonstrate that the populism-Euroscepticism link is contingent, not deterministic; its character is profoundly shaped by ideology, with left-wing and right-wing variants driven by distinct grievances and articulating different visions for Europe's future. Furthermore, the nexus is highly strategic and adaptive, having evolved significantly in the polycrisis environment towards a sophisticated two-level strategy that combines institutional pragmatism with communicative radicalism. This dynamic landscape presents a novel challenge to scholars and policymakers alike, requiring new conceptual tools, innovative research methods, and a comparative empirical foundation to make sense of the forces reshaping the European political order.

The **EUPopLink conceptual framework**, which has emerged from the work of its first Working Group (WG1), is the deliverable that addresses the need for conceptual advancement. As this review has shown, static typologies are insufficient to capture the strategic ambiguity of the contemporary nexus. This publication provides the shared definition and new commonly accepted classifications and typologies necessary to bring clarity, precision, and standardization to future research, creating the robust conceptual toolkit required to analyze the phenomenon in its current, fluid form.

Simultaneously, the forthcoming **EUPopLink series of country reports**, will provide the foundational empirical work needed to push the methodological and empirical frontiers of the field. These reports, by systematically presenting for each country (i) the relevant populist political actors, (ii) a detailed analysis of the populism-Euroscepticism nexus, and (iii) an examination of the responses to Euroscepticism and their consequences, will generate an unprecedented body of comparative data.

This coordinated data collection and analysis is the mechanism through which the supply and demand sides of the nexus can be systematically linked across diverse national contexts. These reports are the vehicle through which the scholarly community will progress beyond the current state of the art, transforming our understanding of one of the most pressing challenges to the future of European integration and liberal democracy.

## Ethics and consent

Ethical approval and consent were not required

## Data Availability

No data associated with this article

## References

[ref-1] Andreadis I Stavrakakis Y Teperoglou E : Unpacking the interplay between populism and Euroscepticism: towards a new operationalization. In: I. Andreadis, Y. Stavrakakis, & E. Teperoglou (Eds.), *Proceedings of the DataPopEU Conference (2022): Populism and Euroscepticism in Perspective.*Sofia PA,2022;123–157. Reference Source

[ref-2] Arce Ó Ciccarelli M Kornprobst A : What caused the euro area post-pandemic inflation? An application of Bernanke and Blanchard (2023). *Occasional Paper Series.* 2024;343. 10.2866/361869

[ref-3] Baute S Abts K Meuleman B : Public support for European solidarity: between Euroscepticism and EU agenda preferences? *J Common Mark Stud.* 2019;57(3):533–550. 10.1111/jcms.12833

[ref-4] Bergmann E : The strategic exploitation of conspiracy theories by populist leaders. *Genealogy.* 2025;9(2):41. 10.3390/genealogy9020041

[ref-5] Carpenter MJ Brunet-Jailly E Hallgrímsdóttir HK : Editorial: crisis, contention, and Euroscepticism. *Front Polit Sci.* 2024;6: 1420335. 10.3389/fpos.2024.1420335

[ref-6] Csehi R Zgut E : ‘We won’t let Brussels dictate us’: Eurosceptic populism in Hungary and Poland. *Eur Polit Soc.* 2021;22(1):53–68. 10.1080/23745118.2020.1717064

[ref-7] Mach Z : Right-wing populism, Euroscepticism, and neo-traditionalism in Central and Eastern Europe. In: *The Right-Wing Critique of Europe.*Routledge,2022. 10.4324/9781003226123-4

[ref-8] Macková AP Čejková L Novotná M : When populism targets Europe: anti-EU rhetoric and user engagement in the visegrád countries. *Media Commun.* 2025;13. 10.17645/mac.10613

[ref-9] Pirro ALP Taggart P : The populist politics of Euroscepticism in times of crisis: a framework for analysis. *Politics.* 2018;38(3):253–262. 10.1177/0263395718770579

[ref-10] Pirro ALP Taggart P van Kessel S : The populist politics of Euroscepticism in times of crisis: comparative conclusions. *Politics.* 2018;38(3):378–390. 10.1177/0263395718770579

[ref-11] Roch J : The populism-Euroscepticism nexus: a discursive comparative analysis of Germany and Spain. Routledge,2023. 10.4324/9781003382744

[ref-12] Rooduijn M van Kessel S : Populism and Euroskepticism in the European Union.In: *Oxford Research Encyclopedia of Politics.* 2019. 10.1093/acrefore/9780190228637.013.1045

[ref-17] Stavrakakis Y Katsambekis G Nikisianis N : Extreme right-wing populism in Europe: revisiting a reified association. *Critical Discourse Studies.* 2017;14(4):420–439. 10.1080/17405904.2017.1309325

[ref-13] van Elsas E van der Brug W : The changing relationship between left–right ideology and euroscepticism, 1973–2010. *Eur Union Polit.* 2015;16(2):194–215. 10.1177/1465116514562918

[ref-14] Vasilopoulou S : European integration and the radical right: three patterns of opposition. *Government and Opposition.* 2011;46(2):223–244. 10.1111/j.1477-7053.2010.01337.x

[ref-15] Vasilopoulou S : The radical right and Euroskepticism. In: J. Rydgren (Ed.), *The Oxford Handbook of the Radical Right.*Oxford University Press,2018. 10.1093/oxfordhb/9780190274559.013.7

[ref-16] Wagner S : Euroscepticism as a radical left party strategy for success. *Party Politics.* 2021;28(6): 13540688211038917. 10.1177/13540688211038917

