# Supplementary material for: The populism-euroscepticism nexus in a contested Europe: The EUPopLink COST Action
Source: Open Res Eur. 2026 Jan 26;6:27. [Version 1] doi: 10.12688/openreseurope.22680.1 (PMC12910195; doi:10.12688/openreseurope.22680.1)
Supplement: Peer review history

# Reviewer report: Reviewer response for version 1

**DOI:** 10.21956/openreseurope.24526.r68890

**Published:** 2026-03-05

**Authors:** Natasza Styczynska

**Affiliations:** 1 Jagiellonian University, Kraków, Poland

The article examines the phenomena of populist Euroscepticism, addressing both its theoretical foundations and its manifestations across Europe. It is important to acknowledge the recognition of the interconnected nature of right-wing and left-wing populist Euroscepticism, which is often overlooked, despite its high relevance. The theoretical discussion reviews current concepts linking populism and Euroscepticism, considers ideological divergence, and situates the debate within the broader polycrisis context.

 The structure and argumentation of the paper are clear and easy to follow. With regard to the literature review, it is recommended that the paper engage more with dimensions of populist Euroscepticism that vary by political position and domestic context. In particular, it would be beneficial to explore the differences between cases where populist Eurosceptics govern(ed) and where they are in opposition. A growing body of case-study and comparative literature (notably on Poland and Hungary, but also Serbia) addresses the activities of the populist in power and could be interesting for the members of the COST network. I am aware of the space constraints that apply to this paper and its introductory nature. The following remarks therefore address the subsequent phases of the project.

Are all factual statements correct and adequately supported by citations?

Yes

Is the review written in accessible language?

Yes

Are the conclusions drawn appropriate in the context of the current research literature?

Yes

Is the topic of the review discussed comprehensively in the context of the current literature?

Yes

Reviewer Expertise:

Euroscepticism, Populism, Political Parties, EU Enlargement

I confirm that I have read this submission and believe that I have an appropriate level of expertise to confirm that it is of an acceptable scientific standard.

# Reviewer report: Reviewer response for version 1

**DOI:** 10.21956/openreseurope.24526.r68897

**Published:** 2026-02-16

**Authors:** Kirk Hawkins

**Affiliations:** 1 Brigham Young University, Provo, UT, USA

This article provides the conceptual and theoretical foundations for a systematic, comparative study of populism and Euroscepticism. While acknowledging various approaches to populism, the authors define this in ideational terms as a discursive frame or thin-centred ideology. The definition allows them to conceptualize Euroscepticism as a distinct type of idea—an issue position--with a contingent relationship to populism. This relationship is dynamic and complex, with multiple sub-types possible on the ideological left and right, and with changes in populist strategy emerging as parties enter government.

 The authors represent the conceptual terrain well and have embraced an approach that is widely accepted in the scholarly literature. Because this article is largely conceptual, the authors are only able to briefly introduce their research design (a series of comparative country studies). This level of detail seems suitable for a conceptual introduction.

 I would be interested in seeing more about their theoretical development. Has the COST action already sketched out a causal argument for the appearance of particular modes of populism (left and right, eurosceptic or not) and for their subsequent impact on democratic stability in certain countries? For now, I assume follows in subsequent Work Packages.

Are all factual statements correct and adequately supported by citations?

Yes

Is the review written in accessible language?

Yes

Are the conclusions drawn appropriate in the context of the current research literature?

Yes

Is the topic of the review discussed comprehensively in the context of the current literature?

Yes

Reviewer Expertise:

Populism

I confirm that I have read this submission and believe that I have an appropriate level of expertise to confirm that it is of an acceptable scientific standard.

# Reviewer report: Reviewer response for version 1

**DOI:** 10.21956/openreseurope.24526.r68888

**Published:** 2026-02-03

**Authors:** Simon Usherwood

**Affiliations:** 1 The Open University, Milton Keynes, UK

The piece provides a clear and well-argued presentation of the central argument on the need for a contingent and dynamic understanding of the link between populism and euroscepticism. It sets out the fundamentals with appropriate referencing of sources, logical argumentation and the logical presentation of the subsequent work packages.

 While it is possible to argue that more detail might be useful, that is neither viable within the constraints of this single piece nor the intention at this stage of the project: the aim is to established a priori a research agenda, which it does very well. As such, there are no recommendations for revisions at this stage.

Are all factual statements correct and adequately supported by citations?

Yes

Is the review written in accessible language?

Yes

Are the conclusions drawn appropriate in the context of the current research literature?

Yes

Is the topic of the review discussed comprehensively in the context of the current literature?

Yes

Reviewer Expertise:

Euroscepticism, EU-UK relations

I confirm that I have read this submission and believe that I have an appropriate level of expertise to confirm that it is of an acceptable scientific standard.
